# Selection for growth rate and body size have altered the expression profiles of somatotropic axis genes in chickens

**DOI:** 10.1371/journal.pone.0195378

**Published:** 2018-04-09

**Authors:** Junjing Jia, Irfan Ahmed, Lixian Liu, Yong Liu, Zhiqiang Xu, Xiaohua Duan, Qihua Li, Tengfei Dou, Dahai Gu, Hua Rong, Kun Wang, Zhengtian Li, Mir Zulqarnain Talpur, Ying Huang, Shanrong Wang, Shixiong Yan, Huiquan Tong, Sumei Zhao, Guiping Zhao, Marinus F. W. te Pas, Zhengchang Su, Changrong Ge

**Affiliations:** 1 Yunnan Provincial Key Laboratory of Animal Nutrition and Feed, Yunnan Agricultural University, Kunming, Yunnan Province, People’s Republic of China; 2 Department of Food Science, Yunnan Agricultural University, Kunming, Yunnan Province, People’s Republic of China; 3 Institute of Animal Sciences, Chinese Academy of Agricultural Sciences, Beijing, People’s Republic of China; 4 Animal Breeding and Genetics, Wageningen UR Livestock Science, Wageningen, The Netherlands; 5 Dali University, Dali, Yunnan Province, People’s Republic of China; 6 Department of Bioinformatics and Genomics, College of Computing and Informatics, the University of North Carolina at Charlotte, Charlotte, NC, United States of America; University of New England, AUSTRALIA

## Abstract

The growth hormone / insulin-like growth factor-1 (GH/IGF-1) pathway of the somatotropic axis is the major controller for growth rate and body size in vertebrates, but the effect of selection on the expression of GH/IGF-1 somatotropic axis genes and their association with body size and growth performance in farm animals is not fully understood. We analyzed a time series of expression profiles of GH/IGF-1 somatotropic axis genes in two chicken breeds, the Daweishan mini chickens and Wuding chickens, and the commercial Avian broilers hybrid exhibiting markedly different body sizes and growth rates. We found that growth rate and feed conversion efficiency in Daweishan mini chickens were significantly lower than those in Wuding chickens and Avian broilers. The Wuding and Daweishan mini chickens showed higher levels of plasma GH, pituitary GH mRNA but lower levels of hepatic growth hormone receptor (GHR) mRNA than in Avian broilers. Daweishan mini chickens showed significantly lower levels of plasma IGF-1, thigh muscle and hepatic IGF-1 mRNA than did Avian broilers and Wuding chickens. These results suggest that the GH part of the somatotropic axis is the main regulator of growth rate, while IGF-1 may regulate both growth rate and body weight. Selection for growth performance and body size have altered the expression profiles of somatotropic axis genes in a breed-, age-, and tissue-specific manner, and manner, and alteration of regulatory mechanisms of these genes might play an important role in the developmental characteristics of chickens.

## Introduction

Growth rate, body size and weight are determined by genotype as well as by environmental factors including nutrition. The neuroendocrine system plays an important role in regulating animal growth. Among all the neuroendocrine factors involved in growth regulation, the hypothalamus–pituitary somatotropic axis has received greatest attention [1]. Studies in human, rodents, and other vertebrate species have shown unequivocally that the growth hormone (GH)/insulin-like growth factor-1 (IGF-1) pathway of the somatotropic axis is the major controller of linear skeletal growth rate and body size [2–10].

GH coordinates postnatal growth of multiple tissues, including skeletal muscles [11]. The growth-promoting actions of GH are mediated by circulating or locally produced IGF-1 [12], which is a critical myogenic agent promoting muscle growth [2,13]. The somatotropic axis consists of GH, upstream hypothalamic hormones, IGF and down-stream signaling molecules. In addition to its direct effects on target tissues, plasma GH can also indirectly exert its functions by stimulating IGF-1 production and secretion in the liver. Both GH and IGF-1 can stimulate the growth of tissues by regulating protein, carbohydrate and lipid metabolisms. Alterations in these interrelated pathways can lead to both growth retardation or tissue proliferation and a variety of metabolic disturbances [2].

Bernard *et al*. examined the effects of genetic selection favoring increased muscle growth on gene expression in the muscles of young bulls [14], and found that many genes of the somatotropic axis were differentially expressed between bulls selected for high growth potential and those for low growth potential. *In vivo* and *in vitro* studies have shown that both IGF-1 and IGF-2 stimulate the proliferation and differentiation of muscle cells through their interactions with the IGF receptors (IGFRs) [15]. Consequently, the somatotropic axis genes can be promising targets for improving meat yield in cattle and pigs [3–6,8,16].

In poultry, numerous studies have been carried out to reveal changes in the somatotropic axis associated with selection and breeding. Mao *et al*. found that different Avian broilers had differential hepatic GH binding activities, but similar steady-state levels of GH receptor (GHR) mRNA [17]. Layers had significantly higher pituitary GH mRNA levels than did Avian broilers at 56 day old, but the difference was not significant at earlier stages [18]. Zhao *et al*. reported that the transcription of the somatotropic axis genes responded differently to nutrition [1]. However, little is known about the relationships between the somatotropic gene expression profiles and the growth performance of chickens, except for the relationship between sex-linked dwarf chickens and the somatotropic axis [19–22]. Therefore, we investigated the relations among body size and growth performance on the one hand and plasma hormone levels and the mRNA expression profiles of the somatotropic axis genes on the other hand. We used a study system differing in body size and growth rate composed of two chicken breeds and a commercial broiler hybrid. The Daweishan mini chickens are a small sized Chinese indigenous Jungle fowl breed with an adult body weight (BW) at 4 months of age of 0.68 kg for hens or 0.92 kg for cocks. The Wuding chickens are a Chinese indigenous chicken breed with an adult BW at 4 months of age of 1.85 kg for hens or 2.20 kg for cocks [23]. The commercial Avian broiler is a modern hybrid selected for fast growth and a large adult BW. At 4 months of age the BW of hens is 4.99 kg, and cock weights 5.30 kg. Indigenous breeds are important for genetic conservation since they may harbor genetic variation lost in commercial hybrids due to selection. Changed gene expression profiles may indicate the biological mechanisms regulated under selection. The objective of this study was to relate the expression profiles of the GH/IGF-1 pathway of the somatotropic axis to growth rate and body size of chickens.

## Materials and methods

### Chicken, diet and housing

All procedures conducted with the chickens had been prior approved by the Yunnan Agricultural University Animal Care and Use Committee (approval ID: YNAU#0006). Animal use and care were in accordance with the Guide for the Care and Use of Laboratory Animals published by the US National Research Council [24].

One day old Daweishan mini chickens and Wuding chickens (local native breeds in Yunnan Province of P. R. China) were purchased from Chicken Farm of Yunnan Agricultural University. Avian broiler chickens of 1 day old were purchased from Kunming Zhengda Group (Kunming, Yunnan, P. R. China).

Twenty chickens from each chicken source were sacrificed at 1 d of age (week 0). A total of 180 chickens were reared under standard conditions on a starter diet to 30 d of age, and then 20 chickens from each were sacrificed (week 4). From 30 d of age on, 40 chickens per chicken source were fed a regular diet and 12.5 MJ /kg Metabolizable Energy) ME) to 60 days (week 8), or 90 days (week 12), and 20 chickens were sacrificed per time point. The diet content was consistent with the formulation to meet NRC 1994 [25] and Chinese Chicken Feeding Standard [26] recommendations. The compositions of diets are shown in Table 1.

**Table 1:** Composition of the period I and period II (g/kg, air dry) used in the experiment.

Dietary Component	Period I ^1^	Period II^1^
**Maize**	591	542.7
**Soy protein**	314.8	297.1
**Soya oil**	20.9	39
**Wheat bran**	-	50
**Fish meal**	30	30
**CaHPO**_**4**_**·2H**_**2**_**O**	18.3	13.3
**Stone meal**	10.8	12
**Lysine**	-	-
**Methionine**	1.5	1.1
**Salt**	2.9	2.7
**Minerals and vitamins mix**^**2**^	10	10
**Metabolizable energy (ME) (MJ/Kg)**	12.1	12.5
**Crude protein (CP)**	200	195
**Crude fat**	55.6	65.6
**Calcium**	9.5	9
**Available phosphorus**	6.8	6.5
**Lysine**	11	10.5
**Methionine + Cysteine**	4.5	4

1: Period I is age 1–30 days; Period II is older than 30 days of age

2: Supplied per kilogram of diet: antioxidant, 100 mg; biotin, 0.3 mg; vitamin A, 12,000 IU; vitamin D3, 3000 IU; vitamin E, 18.75 mg; vitamin K3, 2.65 mg; vitamin C, 12.6 mg; cyanocobalamin, 0.025 mg; folic acid, 2.2 mg; niacin, 35 mg; pyridoxine, 6 mg; riboflavin, 9 mg; thiamine, 3.0 mg; choline chloride, 600 mg; Co, 0.3 mg; Cu, 12 mg; Fe, 50 mg; I, 1 mg; Mn, 125 mg; Mo, 0.5 mg; Se, 200 μg; Zn, 60 mg.

The chickens had free access to feed and water during the entire experimental period. The same temperature and lighting regime was applied in all the experiments, and 1-day-old chickens were kept in floor pens in an environmentally controlled room. The brooding temperature was maintained at 35°C for the first 2 days, and then decreased gradually to 22°C (45% relative humidity) until week 6 and maintained as such to the end of the experiment (week 12). The chickens were allocated randomly to metabolic cages in an enclosed room. During the first 4 weeks of life small groups of animals were joint in the metabolic cages. After week 4 the chicken were housed individually in the metabolic cages. The ambient temperatures varied from 21–24°C and lighting was provided on a light: dark cycle of 12:12h throughout the experiment.

### Growth performance

The BW was measured in the morning, following a 16 h fasting period at every other week starting at 1 d of age. The chickens were weighed in a transport box, which was placed on a tared digital scale (Shanghai Yizhan Weighing Apparatus Limited Company, YZ 0.01g-10kg, China). Feed intakes were recorded daily to calculate the average feed conversion efficiency (FCE) at each time point. The feed intakes were measured as mean for groups for each breed during the first 4 weeks. The feed intakes were measured for each individual animals for all experimental animals after 4 weeks.

### Slaughter procedure

Chickens were sacrificed in accordance with the National Experimental Animal Slaughter Standard of China. Feed was withdrawn 16h and water 12h before slaughter. Chickens were weighed then sacrificed by cervical dislocation.

### Determination of plasma hormones and binding proteins

Blood samples were taken from each chicken via the jugular vein of the neck / wing into vials containing EDTA, and kept on ice. Blood plasma was prepared by centrifugation at 4°C, and stored at -20°C prior to enzyme-linked immunosorbent assay (ELISA). Plasma levels for chicken GH, GHBP, IGF-1, IGFBP2 were measured using an ELISA kit (Shanghai Yeyuan Biotechnology Company, China) and with an ELISA Reader (Thermo Fisher, Varioskan Flash, USA) following the manufacturers’ instructions. Measurements were done in duplicate. Purified chicken GH, GHBP, IGF-1, IGFBP2 antibodies were used to coat microtiter plate wells. The within-run variations in our laboratory were between 4% and 6%, and between-run variations were 5%.

### Expression of somatotropic axis genes

Samples of liver, skeletal muscle (breast muscle and thigh muscle) and pituitary tissues of chickens (N = 20 per time point) were collected and placed in sterile tubes (RNase-free), which were then immediately immersed in liquid nitrogen prior to storage at -80°C until use. Primers (Table 2) were designed to measure the expression of the candidate and reference genes using the software Primer5 (Premier Biosoft international, Palo Alto, CA, USA). Primer specificity was established using the Basic Local Alignment Search Tool (BLAST) from the National Center for Biotechnology Information (http://www.ncbi.nlm.nih.gov/BLAST/). Primers were commercially synthesized (Shanghai Shenggong Biochemistry Company P. R. China). Tissue homogenization, RNA isolation, and RT-qPCR were performed as previously described [27,28].

**Table 2:** Primer sequences for the target genes and their annealing temperatures.

**Genes**	GenBank accession number	Sequence of Primers (5’->3’)	Annealing Temperature (°C)
**18S**	M59389.1	F: CGCGTGCATTTATCAGACCA	58
		R: ACCCGTGGTCACCATGGTA	
**GH**	M35609.1	F: AAGGGATCCAAGCTCCTGAT	54
		R: ATAACCACGTCCCTCAGTGC	
**GHR**	M74057.1	F: CAAGGTGGGAAGAGCACAGT	58
		R: TCCATACTTGGGGTTTCTGC	
**GHBP**	DQ138367.1	F: GGCACTGGTCTGTGCAAAT	60
		R: TCCGGACATTCTTTCCAGTC	
**IGF-1**	NM_001004384.2	F: AGACGAGGCTTCTACTTCA	54
		R: GCAGATTTAGGTGGCTTT	
**IGF1R**	AF041800.1	F: CAGGAACGATGGAGGAGAAG	58
		R: ACGCAAGCAGTGTTGTTGTC	
**IGFBP2**	NM_205359.1	F: CACAACCACGAGGACTCAAA	58
		R: CATTCACCGACATCTTGCAC	

### Statistical analysis

Statistical analyses of growth performance, plasma hormone concentrations and somatotropic axis genes mRNA abundance in tissues among chickens at the same age, and among different ages within a breed / hybrid were performed by the t-test or ANOVA using SPSS 22.0 (IBM Corp, Armonk, NY) software package or normalized data in Excel. Data are expressed as the mean ± SD at each age for each breed. Statistical significance of difference between breeds at an age is labeled by * for P < 0.05, and ** for P < 0.01. Statistical significance of difference between different ages within a breed is labeled by lower-case letters for P < 0.05, and capital letters for P < 0.01.

## Results

### Growth performance

**Fig 1:**
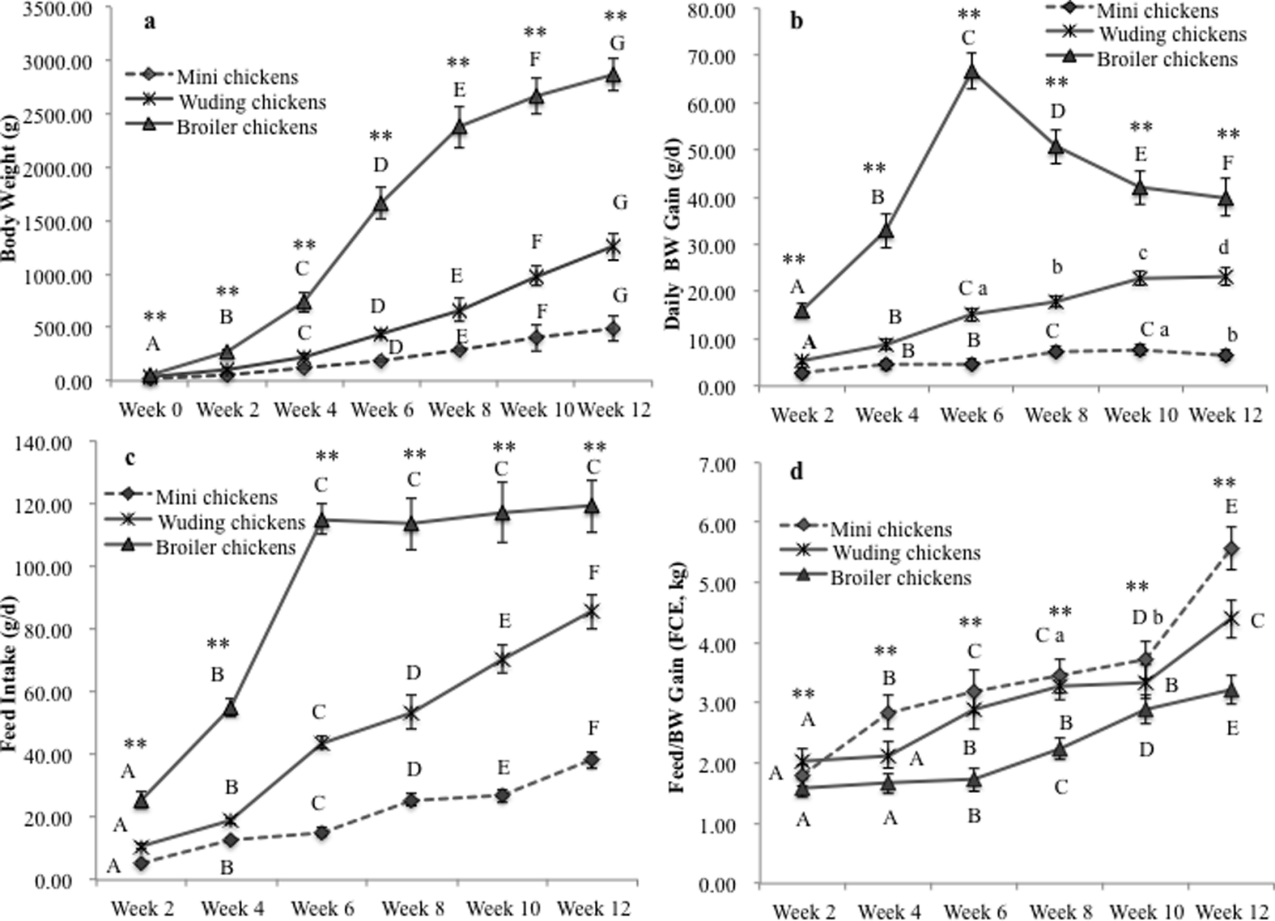
Comparison of growth performance. (a) Body weight (BW), (b) daily BW gain (ADG), (c) feed intake and (d) feed/BW gain (FCE) of the Daweishan mini chicken and the Wuding chicken breeds, and the commercial Avian broiler hybrid chicken hybrid as a function of age from week 0 to week 12, measured every other week. Statistical significance of difference among chickens at a specific age is indicated by asterisks (*: P<0.05; **: P<0.01). Statistical significance among different ages within a breed or hybrid is indicated with letters (lower case: P<0.05; capitals: P<0.01).

As shown in Fig 1, the chickens had significantly different BW, feed intake, average daily BW gain (ADG) and feed/BW gain (feed conversion efficiency, FCE) during the 12-week experiment. The Avian broilers showed the highest initial BW, feed intake and BW gains, while Daweishan mini chickens had the lowest for all these measures except FCE. The Avian broilers approached to the adult BW much faster (Fig 1A) than the two indigenous breeds, while Wuding chickens still showed a slow linear increase in BW and Daweishan mini chickens had an even slower linear increase in BW during the entire experimental period. Consistent with this observation, the Avian broilers showed a sharp peak of growth rate (daily BW gain) at week 6, which then decreased sharply with increasing in age, while Wuding chickens showed a slow linear increase in growth rate and Daweishan mini chickens had an even slower increase in growth rate from weeks 2 to 8, which then decreased with increasing in age (Fig 1B).

As shown in Fig 1C, the Avian broilers showed a sharp increase in feed intake from weeks 2 to 6, followed by a steady state feed intake thereafter. By contrast, both Wuding chickens and Daweishan mini chickens had a linear increase in feed intake during the entire experimental period. All the chickens showed a decrease in FCE (please note: indicated by increasing values, since higher feed intake per BW gain indicates decreasing efficiency) with increasing age (Fig 1D), and the Avian broilers and Daweishan mini chickens had the highest and lowest FCE values (i.e. lowest and highest efficiency), respectively, at each age measured.

### Plasma levels of somatotropic axis components

**Fig 2:**
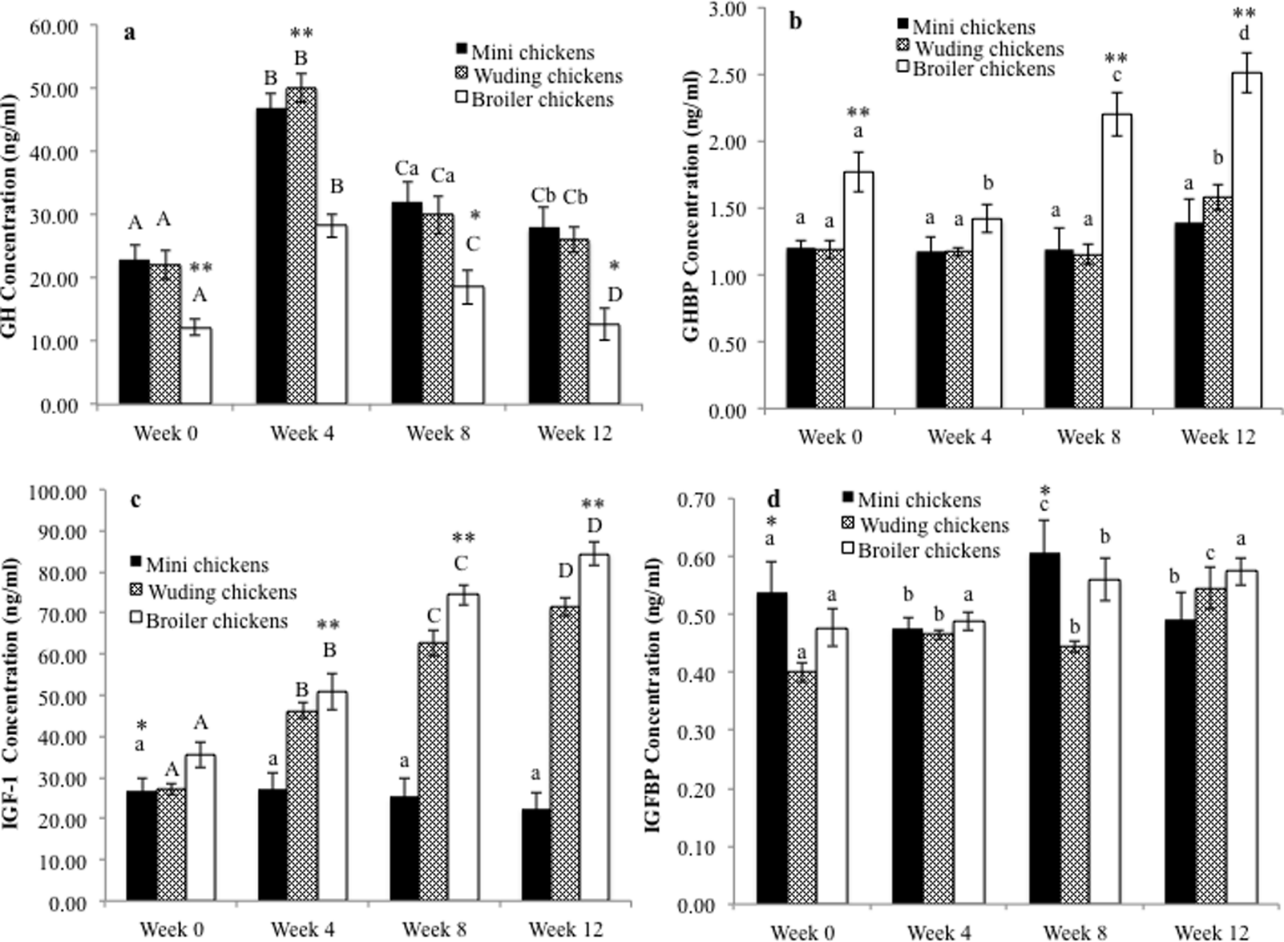
Comparison of plasma hormone concentrations. Plasma concentration of growth hormone (GH) (a), growth hormone binding protein (GHBP) (b), insulin-like growth factor-1 (IGF-1) (c) and insulin-like growth factor-1 binding protein 2 (IGFBP2) (d) of the Daweishan mini chicken and Wuding chicken breeds, and the commercial Avian broiler hybrid at weeks 0, 4, 8 and 12. Statistical significance of difference among chickens at a specific age is indicated by asterisks (*: P<0.05; **: P<0.01). Statistical significance among different ages within a breed / hybrid is indicated with letters (lower case: P<0.05; capitals: P<0.01).

We measured the concentrations of GH, GHBP, IGF-1 and IGFBP2 in the plasma of the chickens at weeks 0, 4, 8 and 12. As shown in Fig 2A, the GH concentrations were significantly lower in the Avian broilers than in Daweishan mini chickens and Wuding chickens at each age measured, while the Daweishan mini chickens and Wuding chickens were similar. All chickens showed the highest plasma GH concentrations at week 4, followed by a decrease with increasing age. The chickens had significantly different plasma GH concentrations at different ages measured, except for Wuding chicken at weeks 0 and 4, and Avian chicken at weeks 4, 8, and 12.

The Avian broilers showed higher plasma GHBP concentrations than the two indigenous breeds, which were similar throughout the experimental period (Fig 2B). Furthermore, the GHBP concentrations were not variable in the two indigenous breeds for the entire experimental period. By contrast, the GHBP concentrations of the Avian broilers increased at the later ages measured.

The Avian broilers showed the highest plasma IGF-1 concentrations while Daweishan mini chickens exhibited the lowest in the 4–12 weeks experimental period (Fig 2C). Interestingly, Daweishan mini chickens maintained similar plasma IGF-1 concentrations throughout the experimental period, while Wuding chickens and the Avian broiler showed a linear increase with increasing age.

Daweishan mini chickens showed the highest plasma IGFBP2 concentrations at weeks 0 and 8 (Fig 2D).

### Expression of GH mRNA in the pituitary, liver and skeletal muscles

**Fig 3:**
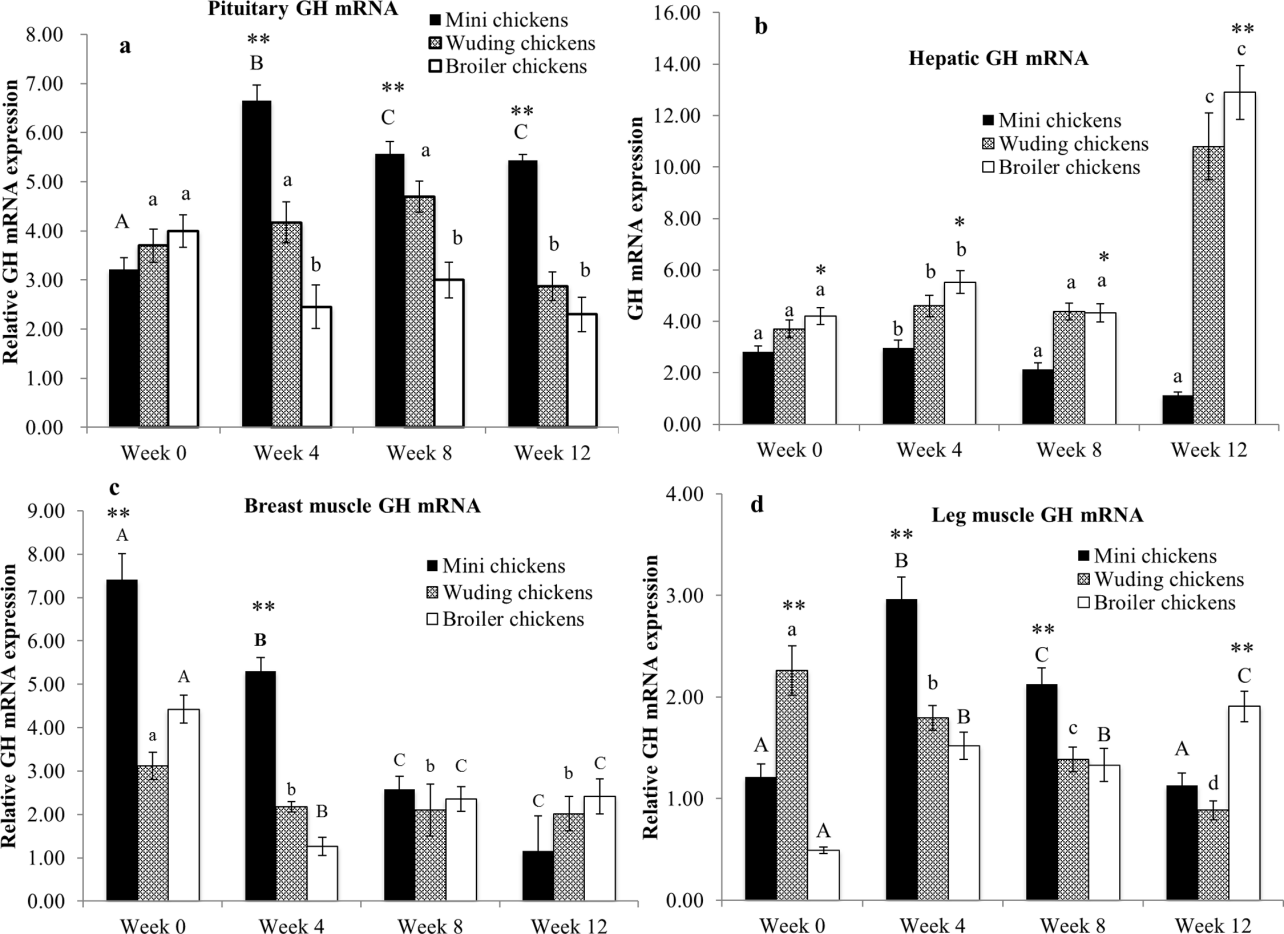
Comparison of growth hormone (GH) mRNA expression. GH mRNA abundance in the pituitary (a), the liver (b), breast muscles (c) and in thigh muscles (d) from the Daweishan mini chicken and the Wuding chicken breeds, and the commercial Avian broiler hybrid at weeks 0, 4, 8 and 12. Statistical significance of difference among chickens at a specific age is indicated by asterisks (*: P<0.05; **: P<0.01). Statistical significance among different ages within a breed / hybrid is indicated with letters (lower case: P<0.05; capitals: P<0.01).

Fig 3A shows that the chickens had similar GH mRNA levels in the pituitary at week 0. Starting from week 4 Daweishan mini chickens and Avian broiler chickens showed the highest and lowest expression levels, respectively. Furthermore, both Daweishan mini chickens and Wuding chickens first displayed an increase in pituitary GH mRNA levels followed a decrease of the expression with increasing age. The Avian broilers had lower pituitary GH mRNA levels at later ages compared with those at week 0.

Both Wuding chickens and the Avian broilers displayed significantly elevated hepatic GH mRNA levels at weeks 4 and 12 (Fig 3B).

As shown in Fig 3C, Daweishan mini chickens showed higher GH mRNA levels in the breast muscles than the two large-sized chicken breeds at weeks 0 and 4, but all the chickens had similar expression levels at weeks 8 and 12. Furthermore, breast muscle GH mRNA levels in all chickens were lower at the later ages than those at week 0.

Daweishan mini chickens showed the highest thigh muscle GH mRNA levels compared with Wuding chickens and the Avian broilers at weeks 4 and 8 (Fig 3D), while Wuding chickens had the highest expression level at week 0. The Avian broilers had the highest expression level at week 12. Thigh muscle GH mRNA levels of Wuding chickens continuously decreased with increasing age. The Avian broilers showed the reverse expression profile. Conversely, thigh GH mRNA levels of Daweishan mini chickens peaked at week 4 and then decreased thereafter.

### Expression of GHR mRNA in the liver and skeletal muscles

**Fig 4:**
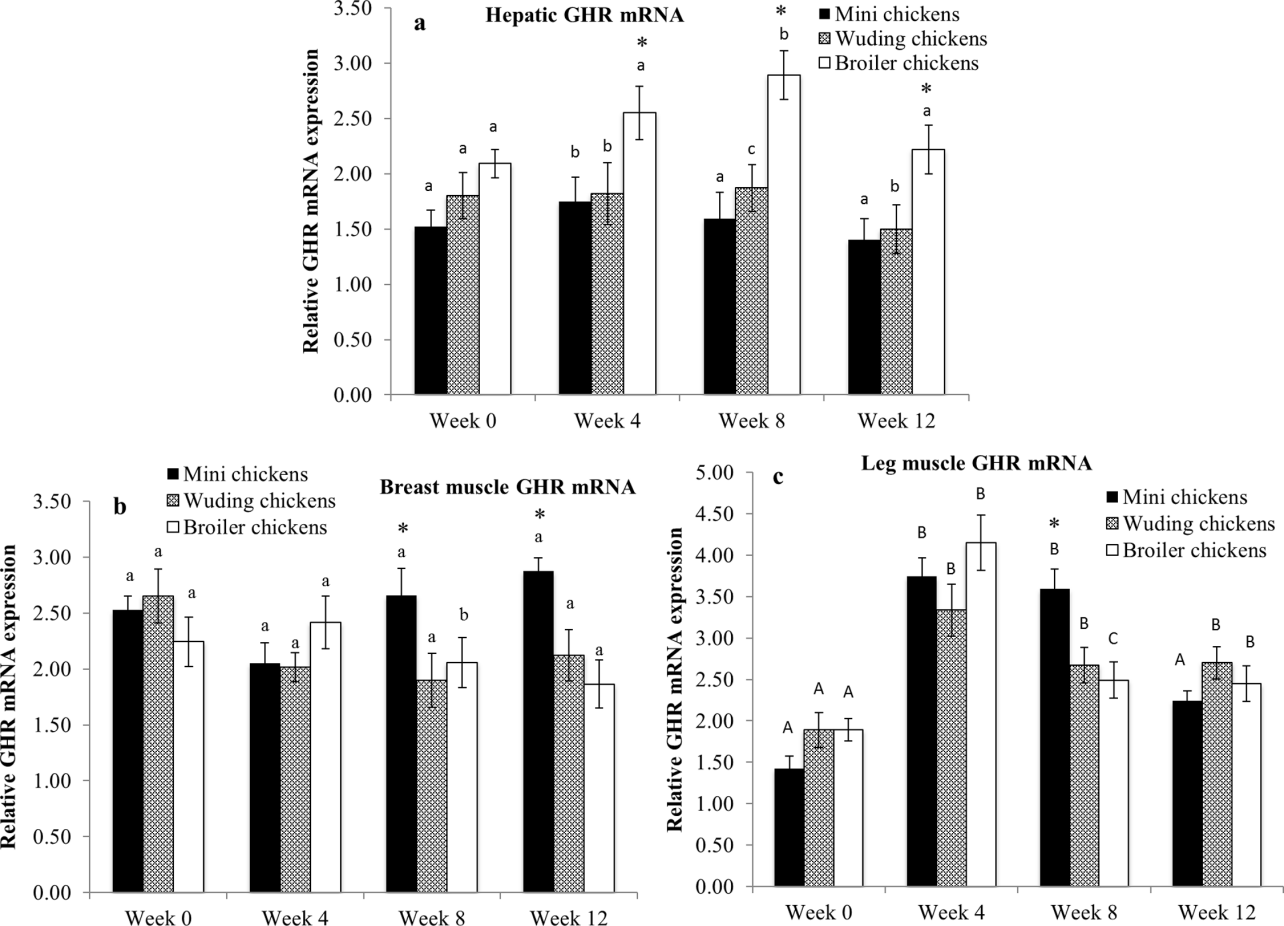
Comparison of growth hormone receptor (GHR) mRNA expression. GHR mRNA abundance in the liver (a), breast muscles (b) and in thigh muscles (c) from the Daweishan mini chicken and the Wuding chicken breeds, and the commercial Avian broiler hybrid at weeks 0, 4, 8 and 12. Statistical significance of difference among chickens at a specific age is indicated by asterisks (*: P<0.05; **: P<0.01). Statistical significance among different ages within a breed / hybrid is indicated with letters (lower case: P<0.05; capitals: P<0.01).

There were no significant differences in the expression of GHR mRNA in the liver at week 0 among the chickens (Fig 4A). The Avian broilers showed the highest GHR mRNA levels in the liver at week 8. All chickens displayed variable expression of GHR mRNA in the liver.

The GHR mRNA levels in the breast muscles exhibited stable expression throughout the experimental period in all chickens (Fig 4B). There were also no significant differences in the GHR mRNA levels in the breast muscles among the chickens at weeks 0 and 4. Daweishan mini chicken showed the highest GHR mRNA levels in the breast muscles at weeks 8 and 12.

The GHR mRNA levels in the thigh muscles of all the chickens peaked at weeks 4 and 8 and then decreased thereafter (Fig 4C). Daweishan mini chickens showed higher expression levels than the Avian broilers and the Wuding chickens at week 8 (*P* < 0.05). There was no significant difference in the expression levels at the other ages measured.

### Expression of GHBP mRNA in the liver and skeletal muscles

**Fig 5:**
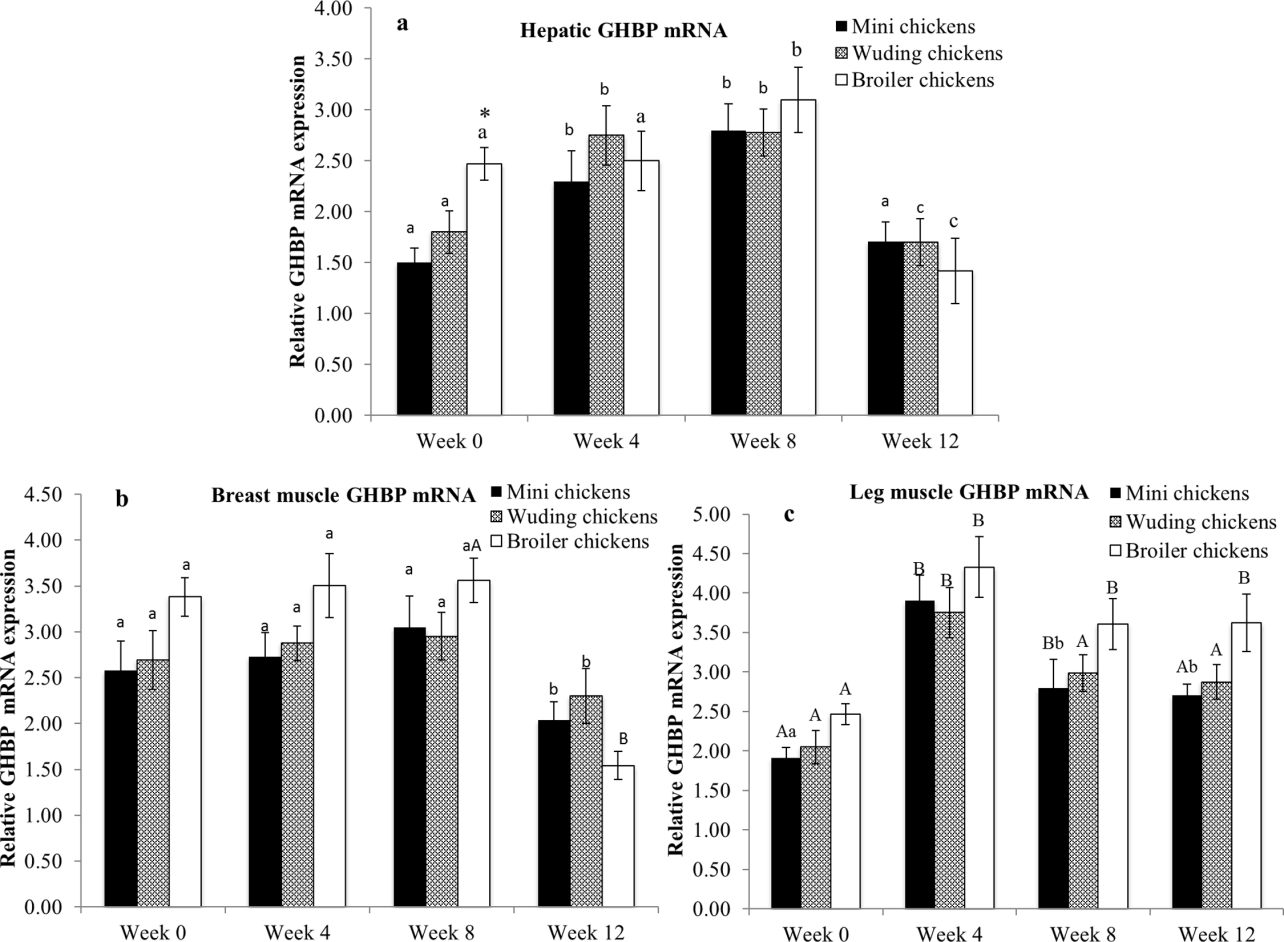
Comparison of growth hormone binding protein (GHBP) mRNA expression. GHBP mRNA abundance in the liver (a), breast muscles (b) and in thigh muscles (c) from the Daweishan mini chicken and the Wuding chicken breeds, and the commercial Avian broiler hybrid at weeks 0, 4, 8 and 12. Statistical significance of difference among chickens at a specific age is indicated by asterisks (*: P<0.05; **: P<0.01). Statistical significance among different ages within a breed / hybrid is indicated with letters (lower case: P<0.05; capitals: P<0.01).

As shown in Fig 5A, the expression of GHBP mRNA showed a peak expression in the liver of the Daweishan mini chickens and the Wuding chickens at week 4, and all the chickens peaked at week 8, followed by a decrease at week 12. Stable GHBP mRNA expression was observed among the chickens except at week 0 when the Avian broilers and Daweishan mini chickens showed high and low expression levels, respectively.

No significant differences were observed in the breast muscles GHBP mRNA levels at all the ages measured (Fig 5B). However, the expression of GHBP mRNA in the breast muscles of all the chickens decreased after week 8.

The GHBP mRNA expression in the thigh muscles showed no breed / hybrid-specific differences of all chickens at all the ages measured (*P* > 0.05) (Fig 5C). The expression of GHBP mRNA in the thigh muscles of all the chickens peaked at week 4 (*P* < 0.05).

### Expression of IGF-1 mRNA in the liver and skeletal muscles

All chickens showed similar expression during weeks 0 and 4. However, the Avian broilers and Wuding chickens showed significantly higher IGF-1 mRNA expression levels of in the liver than the Daweishan mini chickens at weeks 8 and 12 (*P* < 0.01). Furthermore, the Avian broilers and Wuding chickens showed an increase in the expression levels with increasing age during the entire experimental period. On the other hand, Daweishan mini chickens displayed an increase in the expression of IGF-1 mRNA at weeks 0 and 4, followed by a sharp decrease at weeks 8 and 12.

**Fig 6:**
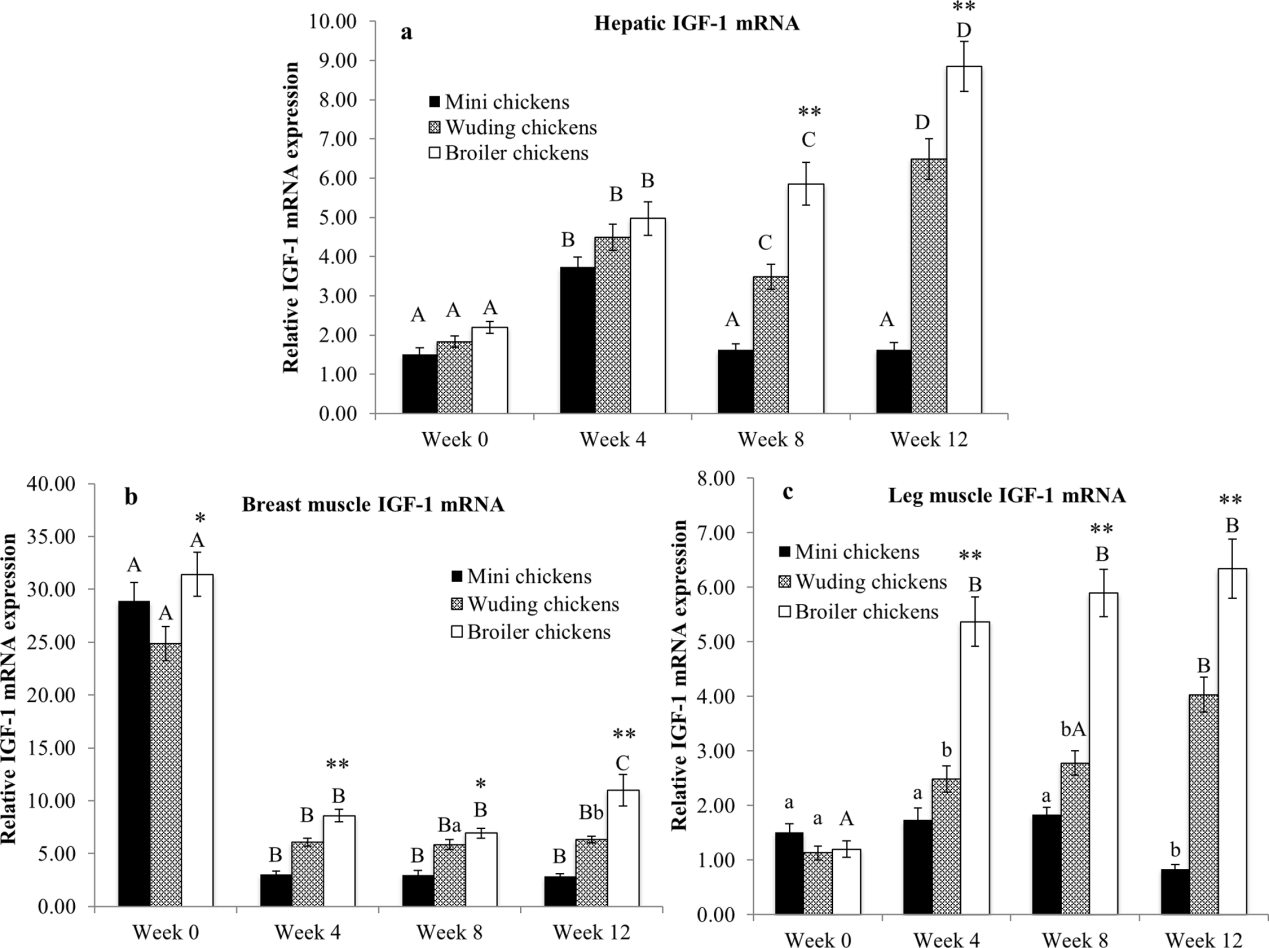
Comparison of insulin-like growth factor-1 (IGF-1) mRNA expression. IGF-1 mRNA abundance in the liver (a), breast muscles (b) and in thigh muscles (c) from the Daweishan mini chicken and the Wuding chicken breeds, and the commercial Avian broiler hybrid at weeks 0, 4, 8 and 12. Statistical significance of difference among chickens at a specific age is indicated by asterisks (*: P<0.05; **: P<0.01). Statistical significance among different ages within a breed / hybrid is indicated with letters (lower case: P<0.05; capitals: P<0.01).

The Avian broilers showed significantly higher IGF-1 mRNA levels in the breast muscles than the two indigenous breeds throughout the experimental period (Fig 6B). On the other hand, all the chickens exhibited relatively high IGF-1 mRNA levels in the breast muscles at week 0, followed by a sharp decrease.

At week 0 the chickens showed similar low thigh muscle IGF-1 mRNA levels. Starting from week 4 the Avian broilers showed highly increased expression levels (Fig 6C). The Avian broilers and Wuding chickens showed elevated IGF-1 mRNA levels after week 0, while the Daweishan mini chickens showed stable expression until week 8 followed by a decrease at week 12.

### Expression of IGF-1R mRNA in the liver and skeletal muscles

**Fig 7:**
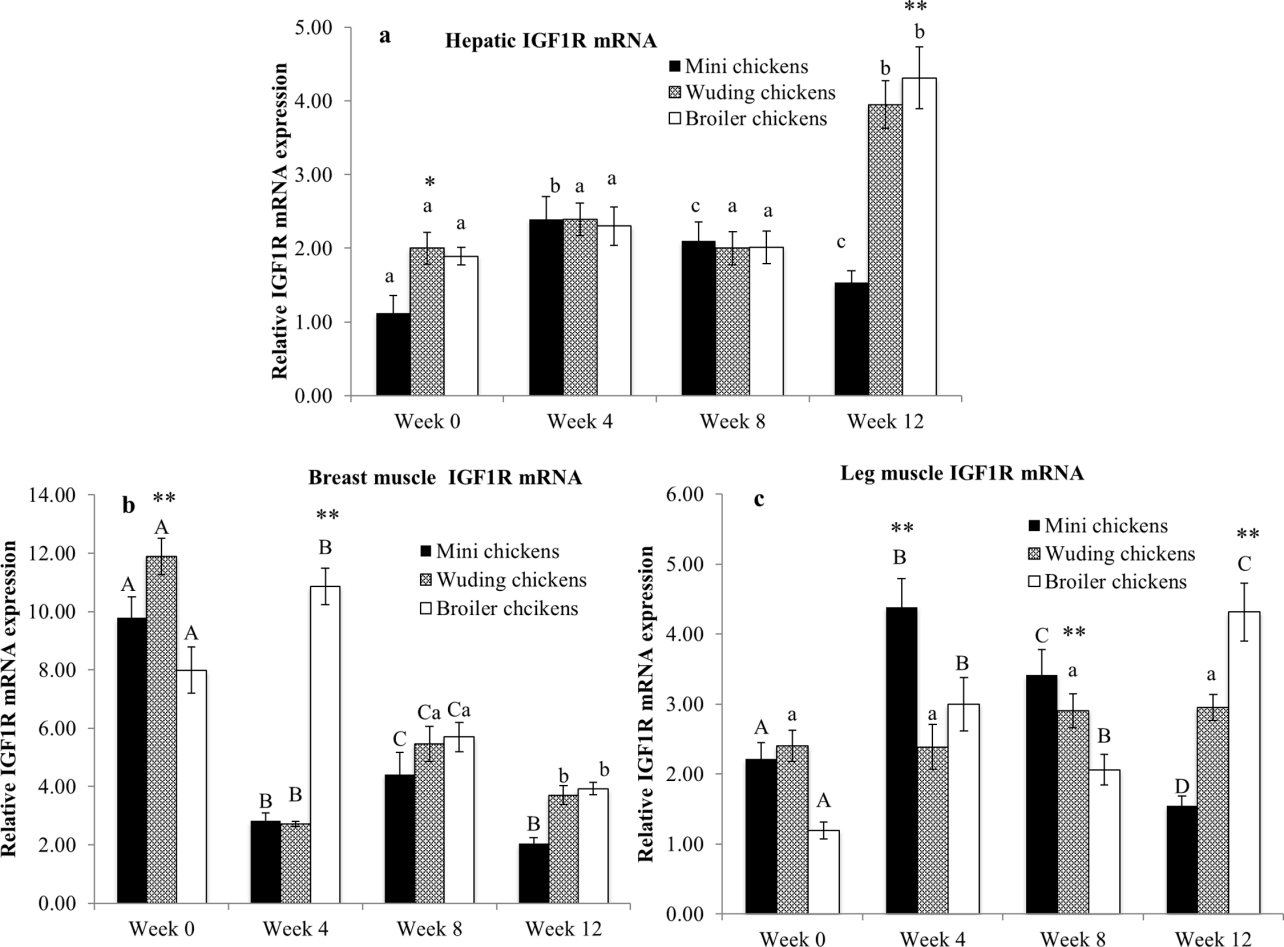
Comparison of insulin-like growth factor-1 receptor (IGF-1R) mRNA expression. IGF1R mRNA abundance in the liver (a), breast muscles (b) and in thigh muscles (c) from the Daweishan mini chicken and the Wuding chicken breeds, and the commercial Avian broiler hybrid at week 0, 4, 8 and 12. Statistical significance of difference among chickens at a specific age is indicated by asterisks (*: P<0.05; **: P<0.01). Statistical significance among different ages within a breed / hybrid is indicated with letters (lower case: P<0.05; capitals: P<0.01).

Fig 7A shows that the hepatic IGF1R mRNA expression was relatively stable from weeks 0 to 8. The Wuding and Avian broilers showed a sharply increased expression in week 12. The Daweishan mini chickens showed a small increase in the expression from weeks 0 to 4 followed by a decrease thereafter.

The IGF-1R mRNA level was overall higher at week 0 compared to the older ages of all chicken in the breast muscles, except for the Avian broiler peak expression at week 4. (Fig 7B).

Fig 7C shows that the Daweishan mini chickens showed a peak expression of the IGF-1R mRNA levels in the thigh muscles at week 4, while the Avian broilers showed peak expression levels at week 12. At week 12 the Avian broilers displayed the highest IGF-1R mRNA level in the thigh muscles while the Daweishan mini chickens showed the lowest as compared with the other chickens. Wuding chickens IGF-1R mRNA levels in the thigh muscles were not significantly different throughout the experimental period.

### Expression of IGFBP2 mRNA in the liver and skeletal muscles

**Fig 8:**
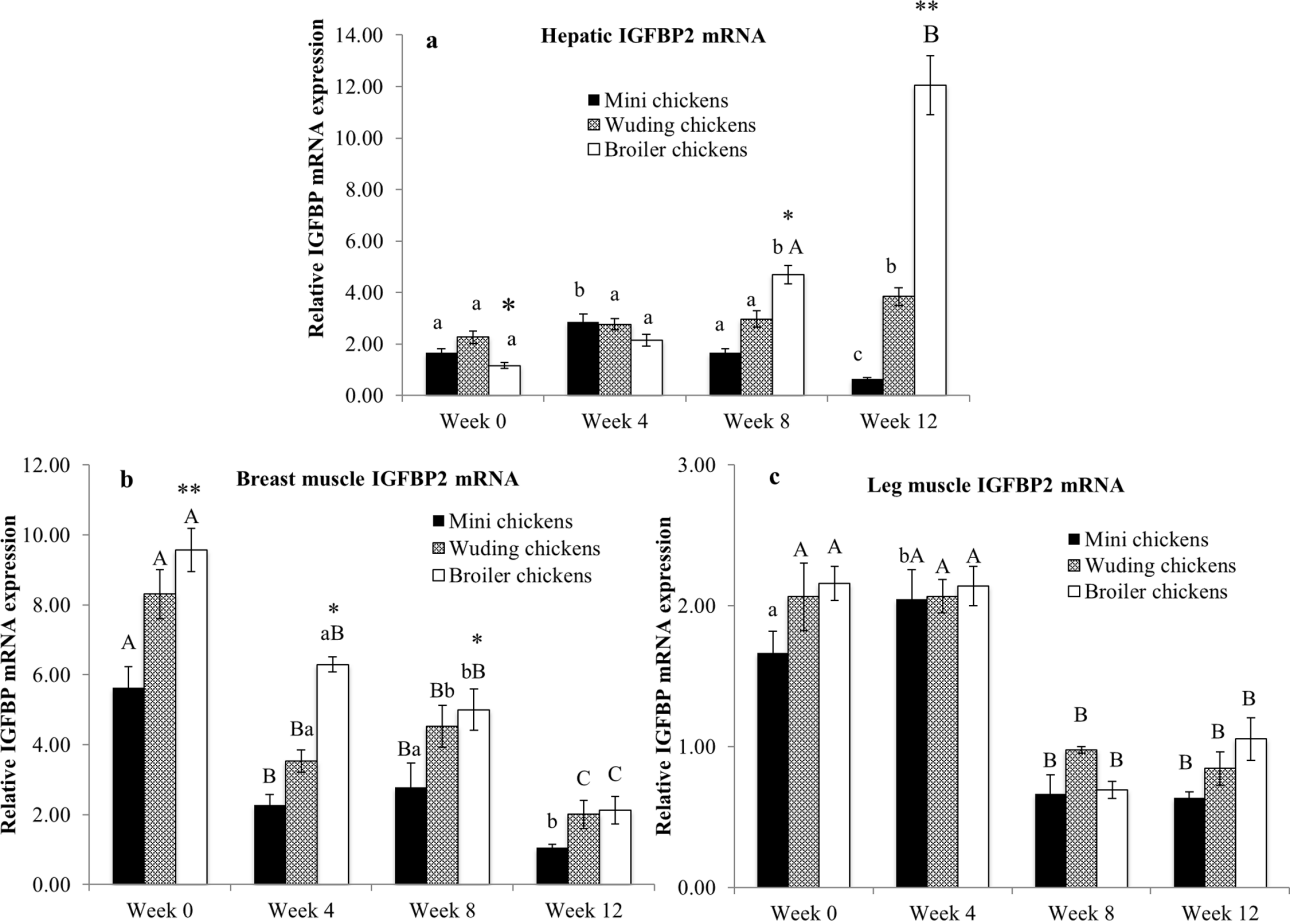
Comparison of insulin-like growth factor-1 binding protein 2 (IGFBP2) mRNA Expression. IGFBP mRNA abundance in the liver (a), breast muscles (b) and in thigh muscles (c) from Daweishan mini chicken and the Wuding chicken breeds, and the commercial Avian broiler hybrid at week 0, 4, 8 and 12. Statistical significance of difference among chickens at a specific age is indicated by asterisks (*: P<0.05; **: P<0.01). Statistical significance among different ages within a breed / hybrid is indicated with letters (lower case: P<0.05; capitals: P<0.01).

Fig 8A shows that the expression level of the hepatic IGFBP2 mRNA is low from week 0 to 8, and sharply increased in Avian broiler chickens at week 12. At the same time the Daweishan mini chickens IGFBP2 expression sharply decreased. Smaller changes included Avian broilers increased expression after week 4, and Wuding chickens increased IGFBP2 mRNA levels in the liver at week 12.

The IGFBP2 mRNA levels in the breast muscles of the Avian broilers and Wuding chickens were significantly higher than those of Daweishan mini chicken throughout the experimental period (Fig 8B). Within each chicken breed or hybrid the IGFBP2 mRNA levels in the breast muscles generally decreased with increasing age.

Fig 8C shows that the expression levels in thigh muscles were similar for all chickens at each time point studied. However, the expression levels decreased after week 4.

### Correlations among the chicken growth performance and somatotropic axis expression patterns

Table 3 shows correlations among the chicken growth performance traits body weight and the expression levels of the somatotropic axis genes. The correlations were separately calculated per tissue. Pituitary GH expression levels correlated negatively with growth traits. Pituitary GH expression correlated positively with IGF1, IGF1R, and IGFBP2 expressions in the liver and breast muscle, while correlations in the thigh muscle were not significant. The GH receptor expression levels showed non-significant correlation profiles in the liver and both muscles. The hepatic GHBP expression levels correlated with production traits only in the muscle tissues. The hepatic GHBP expression levels correlated reversely to GH and its receptor.

**Table 3:** Correlations between growth performance and somatotropic axis gene expression levels in liver, skeletal muscle and pituitary.

	BW	ADG	Feed/gain	GH	GHR	GHBP	IGF1	IGF1R	IGFBP
**Liver**									
BW	1	0.247	0.152	0.701*	0.428	-0.025	0.841**	0.626*	0.848**
ADG		1	-0.378	0.758**	0.509*	0.242	0.516*	0.236	-0.005
Feed/gain			1	0.050	-0.472	-0.313	0.006	0.162	0.029
GH				1	0.256	-0.257	0.870**	0.897**	0.817**
GHR					1	0.524*	0.468	0.298	0.319
GHBP						1	0.021	-0.07	-0.193
IGF1							1	0.836**	0.840**
IGF1R								1	0.761**
IGFBP									1
**Breast muscle**									
BW	1	0.247	0.152	-0.382	-0.357	-0.271	-0.293	-0.246	-0.318
ADG		1	-0.378	-0.534*	-0.317*	0.649*	-0.122	0.326	0.261
Feed/gain			1	-0.425	0.454*	-0.567*	-0.566*	-0.654*	-0.743*
GH				1	0.012	0.019	0.619*	0.282	0.312
GHR					1	0.005	0.032	0.079	0.084
GHBP						1	0.097	0.393	0.560*
IGF1							1	0.714*	0.800**
IGF1R								1	0.821**
IGFBP									1
**Thigh muscle**									
BW	1	0.247	0.152	-0.082	-0.423*	0.404	0.870**	0.301	-0.471
ADG		1	-0.378	-0.345	-0.372	0.557*	0.626*	-0.096	0.208
Feed/gain			1	-0.079	0.472	-0.073	-0.124	0.089	-0.554*
GH				1	0.385	0.295	-0.045	0.689*	0.166
GHR					1	0.680*	0.185	0.495	0.240
GHBP						1	0.620*	0.474	0.088
IGF1							1	0.360	-0.266
IGF1R								1	-0.031
IGFBP									1
**Pituitary**									
BW	1	0.247	0.152	-0.517*					
ADG		1	-0.378	-0.476*					
Feed/gain			1	0.244*					
GH				1					

*: Significance of correlation at P<0.05

**: Significance of correlation at P<0.01

The hepatic IGF1 group expression consisting of IGF1, IGF1R and IGFBP2 correlated well with BW. No correlation was found in the breast muscle, and only IGF1 expression levels correlated with growth traits in the thigh muscle. The IGF1 group correlated with FCE in the breast muscle, which was also significant for IGFBP2 in the thigh muscle. Correlations between GH and the IGFs are as expected.

Correlations with plasma ELISA data were not included in the Table. All calculated correlations among plasma protein concentrations were highly significant (close to 0.99). The plasma concentrations correlated negative with growth rate and BW.

## Discussion

We conducted a comprehensive analysis of the expression profiles of the genes of the somatotropic axis in four tissues and plasma in chicken differing in growth rate and BW. The somatotropic axis is well known. The biological effects of the GH and IGF’s is regulated by their receptors and binding proteins. However, the entire biological mechanism includes connections with several other signaling mechanisms as well [29]; http://sabiosciences.com/pathway.php?sn=Growth_Hormone_Signaling; https://www.wikipathways.org/index.php/Pathway:WP2657). The interested reader is referred to this review and the pathways databases.

y, which is consistent with Both Chinese indigenous chicken breeds showed higher plasma GH levels than did the Avian broilers at all ages examined. While the indigenous Chinese breeds differ in body size they both have a slower growth rate than the Avian broilers. Thus, our results are in agreement with earlier reports that slow growing layer chickens displayed a higher plasma GH level than did fast growing Avian broiler chickens [1]. It has also been shown that the Avian broiler had consistently lower plasma GH levels than a hybrid produced for better feed conversion rate [30]. Interestingly, both Daweishan mini chickens and Wuding chickens consistently had lower plasma GHBP levels than did the Avian broiler at all ages examined. Since GH levels were strongly inversely correlated with GHBP levels, the higher plasma GH levels in Daweishan mini chickens and Wuding chickens might be due to their lower plasma GHBP levels. Although the biological role of GHBP remains unclear, the binding protein can influence GH bioactivity and action kinetics [31].

It has been shown that the expression of GH mRNA in the pituitary and liver [18,32] and the expression of GHR mRNA in the liver exhibit different ontogenetic patterns in different chicken breeds [17,33]. Slow growing layer chickens displayed higher levels of GH mRNA in the pituitary but lower levels of GHR mRNA in the liver, similar to our two slow growing Chinese indigenous breeds, while the opposite was true for fast growing Avian broiler chickens [1]. These results may indicate that GH is differently regulated in different tissues indicating different roles of the hormone. This may be caused also by the selection background of the breeds. Compared to the fast-growing Avian broilers with a large adult BW, both the slow growing Wuding chickens with a large adult BW and Daweishan mini chickens with a small adult BW showed higher levels of plasma GH and pituitary GH mRNA, but lower levels of hepatic GHR mRNA, suggesting that growth rate rather than body size is regulated by this part of the somatotropic axis. Furthermore, this negative correlation is in agreement with the relation between plasma GH levels and hepatic GH binding activity in chickens [1,34–36], and might be caused by the down-regulation of hepatic GH receptors by elevated plasma GH levels [17]. It is therefore likely that the slow growing Daweishan mini chickens and Wuding chickens have lower hepatic GH binding activities compared with the fast-growing Avian broilers. Thus, the selection for high growth rate in Avian broiler chickens might have increased hepatic GH binding activity. Since this mechanism regulates among others the synthesis of IGF-1 –and thereby growth rate–this may be the underlying regulatory mechanism for elevated growth rate in the Avian broiler breed.

It has been shown that selection for fast muscle growth results in changes in gene expression profiles in muscles of young bulls [14]. Many genes of the somatotropic axis were differentially expressed between bulls with different growth potentials [16]. It has been reported that GHR mRNA levels in *longissimus dorsi muscles* (LM) in slow growing pigs were up-regulated more slowly than in fast growing pigs [37,38]. In the present study, we found that both slow growing Daweishan mini chickens and Wuding chickens showed higher skeletal muscle GHR mRNA levels than did the fast-growing Avian broilers. In line with this observation, it has been shown that hepatic GHR mRNA levels in Avian broiler chickens and layer chickens were inversely related to pituitary GH mRNA levels, but positively related to BW within lines, and that layer chickens showed a higher GHR mRNA level in muscles than did Avian broiler chickens [1,30], again suggesting that growth rate rather than body weight determined the selection response of the somatotropic axis. We observed that the levels of plasma GH and skeletal muscle GHR mRNA were negatively correlated with ADG, growth performance and FCE, which is in agreement with earlier findings [16,37–39]. In addition, Castigliego *et al*. [40] reported that the expression of GHR in muscles was down-regulated in bovine after administration of recombinant bovine GH. Spurlock *et al*. found that administration of clenbuterol, a β_2_-adrenergic receptor agonist, resulted in increased BW, and decreased expression of muscle GHR in mice [39], suggesting that different biological stimuli can act via the same biological mechanism.

GHBP is derived from proteolytic cleavage of the extracellular domain of GHR, and therefore, the expression levels of GHR in tissues can be reflected by the plasma levels of GHBP [41]. The Daweishan mini and Wuding chickens consistently showed significantly lower plasma GHBP levels and lower hepatic GHR mRNA levels than the Avian broilers, and had similar age-dependent expression of GH mRNA, GHR mRNA and GHBP mRNA. These results again point to the importance of growth rate over body weight as selection response for the somatotropic axis.

Regarding to the IGF-1, IGF1R and IGFBP2 pathway, we observed that slow growing Daweishan mini chickens with a small BW showed significantly lower levels of plasma IGF-1, thigh muscle and hepatic IGF-1 mRNA, and hepatic GHR mRNA compared to the large-sized Avian broilers and Wuding chickens. Thus, body weight may be regulated by the IGF1 part of the somatotropic axis. Reduced GHR expression might also contribute to the decreased expression of IGF-1 in Daweishan mini chicken since it has been reported that dwarf chicken lacking the GHR gene, had low levels of circulating IGF-1 [42] and that sex-linked dwarf chickens showed a significant lower level of IGF-1 mRNA in tissues compared with normal chickens [43]. Our results are in agreement also with reports that animals having heavier carcasses with greater LM area showed a significantly high level of IGF-1 mRNA compared with animals producing lighter carcasses [16,44]. The expression of IGF-1 was also lower in the LM tissue of growth-restricted piglets compared with control animals [45]. Serum IGF-1 concentration and the IGF-1 mRNA expression in the liver were positively correlated to the growth performance of pigs [45,46]. Furthermore, IGF-1 mRNA expression is positively associated with increased body weight as demonstrated by transgenic over-expression of IGF-1 in mice. Over-expression of IGF-1 at 1.5-fold the normal levels resulted in a 30% increase in body weight [47]. We conclude that indeed IGF-1 seems to be the direct regulator of growth rate and body weight, with GH acting as an opposing regulatory factor for growth rate. However, it is likely that GH levels are inversely related to IGF1 levels because of negative feedback of circulating IGF1 on the GH cells in the pituitary. The increased GHBP levels in fast-growing chickens may also be a consequence of high IGF1 levels, resulting (potentially) in a similar decrease of GH function like negative feedback on synthesis/secretion does.

Serum IGFBPs levels were negatively related to ADG in pigs [46]. Similarly, we found that Daweishan mini chickens had significantly higher levels of plasma IGFBP2 than did the Avian broilers and Wuding chickens before week 8, and the plasma IGFBP2 level was negatively related to BW and ADG. Skeletal muscle IGFBP2 mRNA levels were also significantly higher in Daweishan mini chickens than in the Avian broilers and Wuding chickens during weeks 0–8. Further, skeletal muscle IGFBP2 mRNA levels were positively related to plasma IGFBP-2, but negatively related to hepatic IGFBP2 mRNA levels, BW, ADG and FCE. These findings are in agreement with results that (1) muscle IGFBP levels were significantly lower in gilts bred for enhanced growth performance [3–6,46], (2) hepatic IGFBP expression was higher in slow growing breeds than in fast-growing breeds of pigs [48], and (3) hepatic IGFBP expression was negatively related to ADG and 9-wk BW in average nursery pigs [49]. Thus, it is likely that binding of IGFBP to IGF-1 might inhibit the action of IGF-1 in these animals. We found that Daweishan mini chickens showed a higher plasma IGFBP2 level and a higher IGFBP2 mRNA level in the liver, but lower IGF-1 and IGF-1R mRNA levels in the liver compared to Wuding chickens and the Avian broilers, suggesting a relation with body size. Therefore, it is possible that plasma IGFBPs may compete with IGF-1R for binding to IGF-1, thereby altering the equilibrium between IGF-1 and IGF-IR in regulating IGF-1 effects [50]. Indeed, it has been reported that overexpression of IGFBP isoforms inhibits IGF-1 action by inhibiting its binding to IGF-1R [51]. In addition, during the development of Avian broiler chickens, IGFBP2 mRNA in epiphyseal cartilage transiently reaches a peak between embryonic day 12 and 18, and gradually decreases by 42 weeks after hatching, suggesting that IGFBP-2 may negatively affect tibia growth after hatching [52]. In this study, we found exactly the same pattern of age-related expression of IGF-1 mRNA, IGF-1R mRNA and IGFBP mRNA between the Wuding chickens and the Avian broilers. Thus, the IGF-1, IGF-1R and IGFBP pathway also plays a crucial role in growth and BW in chickens.

In conclusion, the results from this study further strengthen the notion that selection for growth performance, BW and size has resulted in changes in the expression profiles of the somatotropic genes in chickens. Consequently, the somatotropic axis genes and their regulatory sequences are likely to be promising candidate biomarkers to understand selection response for growth rate and meat yield, and to improve these traits in chickens.
